# Mental Capacity Assessments on Admission: An Audit

**DOI:** 10.1192/bjo.2026.11564

**Published:** 2026-06-30

**Authors:** Farah Ahmed, Charlotte Forbes

**Affiliations:** Devon Partnership Trust, Torquay, United Kingdom

## Abstract

**Aims:**

To assess the quality of mental capacity assessments completed on admission and compliance with Devon Partnership Trust’s Inpatient Admissions Standard Operating Procedure. This requires that each patient admitted to a mental health ward has an assessment of capacity to consent to treatment completed within 24 hours of admission. In line with the Mental Capacity Act (MCA) Code of Practice, capacity assessments should be decision-specific, include the four-part functional test of mental capacity, represent the least restrictiveoption and be made in the patient’s best interests where capacity is lacking.

**Methods:**

Retrospective audit of clinical records for the most recent 30 patients admitted to the Beech Unit in Torbay, Devon until December 2025. Records were assessed according to the following audit criteria:

1. Assessment of capacity to consent to admission and treatment documented within 24 hours of admission

2. Nature of decision clearly documented

3. Use of the four-part functional test (ability to understand, retain, use or weigh up and communicate a decision)

4. Reference to least restrictive practice and best interests where patients lacked capacity

**Results:**

Every patient had a mental capacity assessment documented within 24 hours of admission (100%). The standardised MCA template (which prompts clinicians to document all parts of a capacity assessment) was completed for 90% of the patients and only half were completed in the timeframe. 90% of the records clearly documented the decision in question and 90% had clear documentation of the functional test. Fewer records contained the direct statements ‘least restrictive’ and ‘best interests’ (53%).

**Conclusion:**

All patients (100%) had an assessment of mental capacity documented following admission to the ward, showing that clinicians have a good understanding of when the Mental Capacity Act should be considered, but timeliness of completing the MCA template could be improved. While the nature of decisions and use of the four-part test werewell documented overall, documentation around recording of best interests and least restrictive options could also be improved.

There are some limitations to this audit: variability in clinician documentation styles may affect interpretation of the data and retrospective data collection limits clarification of clinical reasoning.

Recommendations include targeted interventions to guide clerking doctors about when to complete MCA assessments and what should be included. It is recommended that the Trust’s standardised MCA template is used to improve the quality of documentation, as this prompts clinicians to address the key elements of a good capacity assessment.

